# Sinapine Thiocyanate Inhibits the Proliferation and Mobility of Pancreatic Cancer Cells by Up-Regulating GADD45A

**DOI:** 10.7150/jca.65212

**Published:** 2022-01-24

**Authors:** Jingya Wang, Zhirui Zeng, Shan Lei, Junbin Han, Shanggao Liao, Jinjuan Zhang, Lu Wang, Yuhua Dong, Haiyang Li, Tengxiang Chen

**Affiliations:** 1 Guizhou Provincial Key Laboratory of Pathogenesis & Drug Research on Common Chronic Diseases, Department of Physiology, School of Basic Medical Sciences, Guizhou Medical University, Guian New District 550025, Guizhou, People's Republic of China.; 2 Department of Surgery, Affiliated Hospital of Guizhou Medical University, Guiyang 550009, Guizhou, People's Republic of China.; 3 Guizhou Institute of Precision Medicine, Affiliated Hospital of Guizhou Medical University, Guiyang 550009, Guizhou, People's Republic of China.; 4 Institute of Radiation Medicine, Fudan University, Xietu Road 2094, Shanghai 200032, People's Republic of China.; 5 School of Pharmacy, Guizhou Medical University, Guian New District 550025, Guizhou, People's Republic of China.

**Keywords:** Sinapine thiocyanate (ST), growth arrest and DNA damage inducible alpha (GADD45A), Pancreatic cancer (PC)

## Abstract

**Background:** Sinapine thiocyanate (ST), an alkaloid isolated from the seeds of cruciferous species, has exhibited anti-inflammatory, anti-malignancy, and anti-angiogenic effects in previous studies. However, the effects and molecular mechanisms of action of ST in pancreatic cancer (PC) are still limited.

**Materials and methods:** PC cells were treated with different concentrations (0, 20, 40, and 80 μM) of ST. The proliferative ability of PC cells *in vitro* was determined using cell count kit-8 (CCK-8), 5-ethynyl-2ʹ deoxyuridine, colony formation, and flow cytometry assays. The mobility of PC cells *in vitro* was analyzed using wound healing assay, transwell assay, Western blotting, and immunofluorescence. High-throughput sequencing followed by bioinformatics analysis, reverse-transcriptase quantitative polymerase chain reaction (RT-qPCR), and Western blotting were performed to identify the key targets of ST. Finally, CCK-8 assay, wound healing assay, and xenograft tumor model were used to determine the relationship between ST and growth arrest and DNA damage-inducible alpha (GADD45A; the key target of ST) and malignant biological properties of PC *in vitro* and *in vivo*.

**Results:** ST significantly repressed the PC cell proliferation rate and colony formation *in vitro* and arrested cells in the G2/M phase. ST inhibited PC cell mobility *in vitro* and increased E-cadherin expression (an epithelial biomarker). GADD45A was considered the key target of ST in PC and was elevated in PC cells treated with ST. The inhibition of GADD45A significantly alleviated the suppressive effects of ST on PC cell proliferation and mobility *in vitro*. ST suppressed PC cell proliferation *in vivo* and increased GADD45A expression in tumor tissues.

**Conclusion:** ST exhibited significant anti-tumor effects on PC cells by upregulating GADD45A. ST may be a potential drug for PC treatment.

## Introduction

Pancreatic cancer (PC) is the twelfth most common malignancy and the seventh main cause of cancer mortality worldwide 1. Although considerable effort has been made to understand the molecular mechanisms of the disease and to develop therapy, patient survival rate is significantly low, (5-year survival rate: <8%) 2. PC treatment is a significant challenge for clinicians because PC is extremely aggressive 3. Similarly, because of the low response rate to radio-chemotherapy and lack of effective biomarkers, the therapy for PC is low success 4. Therefore, there is an urgent need to explore novel therapeutic drugs and effective therapeutic targets for PC.

Growth arrest and DNA damage-inducible alpha (GADD45A) mediates the response to physiological and environmental stress by regulating cell cycle arrest, cell survival, and apoptosis and acts as a tumor suppressor in a series of cancers 5,6. A study demonstrated that the high promoter methylation and low transcription of GADD45A were positively associated with poor prognosis in patients with PC 7. Similarly, another study demonstrated that exogenous transfection of GADD45A decreases PC cell proliferation and induces cell apoptosis by activating the P53 pathway 8. Additionally, evidence suggests that several drugs with anti-tumor activity based on GADD45A upregulation can induce cell apoptosis in some tumor cells 9. Therefore, the induction of GADD45A upregulation is a strategy for PC treatment.

Sinapine thiocyanate (ST) is an active compound found in the seeds of cruciferous species. ST has various pharmacological effects such as anti-inflammatory 10, antioxidant 11, and anti-angiogenic effects 12. Liu* et al.* revealed that ST protects vascular endothelial function and alleviates vascular endothelial cell injury in patients with spontaneous hypertension by repressing NLR family pyrin domain containing 3 inflammasome activation and reducing the expression of inflammatory mediators 13. However, the effects and mechanisms of ST in PC have not been clarified.

This study demonstrated that ST treatment upregulates GADD45A, arrests G2/M phase, and represses epithelial-mesenchymal transition (EMT) repression and thus, leads to the inhibition of PC cell proliferation and migration. This evidence unveils a novel mechanism of ST in anti-PC activity and provides a promising strategy for PC treatment.

## Materials and Methods

### Cell culture, drug, and transient transfection

Human pancreatic cell lines (PANC-1, MIA PaCa-2, and AsPC-1) and hepatocellular carcinoma cell lines (HepG2, Hep3B, and Huh7) were obtained from the American Type Culture Collection (USA). The normal human pancreatic epithelial cells were obtained from Procell Life Science & Technology Co., Ltd. (Wuhan, China). Cholangiocarcinoma cell lines (TFK-1 and RBE) were obtained from the BeNa Culture Collection (Beijing, China). PANC-1, MIA PaCa-2, HepG2, Hep3B, Huh7, TFK-1, and RBE cells were cultured in DuIbecco's modified eagIe's medium (DMEM; Thermo Fisher Scientific, USA) with 10% fetal bovine serum (FBS; Invitrogen, USA) at 37℃ with 5% CO_2_, whereas AsPC-1 and normal human pancreatic epithelial cells were cultured in Roswell Park Memorial Institute (RPMI)-1640 medium with 10% FBS at 37℃ with 5% CO_2_. ST and nocodazole were obtained from MCE (HY-N0450 and HY-13520; Wuhan, China) and dissolved in dimethylsulfoxide (DMSO). GADD45A small interfering RNA (siRNA; si-GADD45A) and corresponding nonsense control siRNA (NC) were purchased from GeneCopoeia (Guangzhou, China). Transient transfection was performed as follows: PC cells were seeded in 6-well plates and transfected with siRNAs using Lipofectamine 2000 (Invitrogen, Carlsbad, CA, USA) after cells reached 50% confluence. The transfection efficiency in PC cells was determined by Western blotting after incubation at 37℃ with 5% CO_2_ for 48 hours. The sequence of si-GADD45A was 5'- GGAGGAAGUGCUCAGCAAA-3', and the sequence of the NC siRNA was 5'-AAAAACGGTAGATGCATCAGC-3'.

### Cell count kit-8

PC cells were seeded in 96-well plates (3 × 10^3^ cells/well) and incubated with different concentrations (0, 20, 40, and 80 μM) of ST. After 24 and 48 hours, 10 μL cell count kit-8 (CCK-8) reagent (Boster; Wuhan, China) was added to each well. The proliferative rates of cells were determined using a spectrophotometer at 450 nm wavelength.

### 5-Ethynyl-2ʹ deoxyuridine assay

5-Ethynyl-2ʹ deoxyuridine (EdU) assay was performed using the Yefluor 488 EdU Imaging Kit (Yeasen, China) according to the manufacturer's instructions. Briefly, PANC-1, MIA PaCa-2, and AsPC-1 cells were cultured in an imaging-appropriate dish and treated with DMSO and ST. Thereafter, cells were incubated with 50 μM EdU reaction reagent for 2 hours, washed with phosphate-buffered saline (PBS) two times, immobilized with 4% paraformaldehyde for 30 min, and incubated with Apollo staining reaction solution. Nuclei were stained with 4,6-diamino-2-phenylindole and visualized using a fluorescence microscope at 488 nm (magnification 400×).

### Colony formation assay

A total of 1.5 × 10^3^ PC cells were seeded in six-well plates and treated with different concentrations (0, 20, 40, and 80 μM) of ST. After culturing for 10 days, cell colonies were immobilized with 4% paraformaldehyde and stained with 0.5% crystal violet (Boster, Wuhan, Boster). Thereafter, a stereogram and micrograph of the colony plates were obtained using a camera and optical microscope (magnification 40×), respectively.

### Flow cytometry analysis

The change in cell cycle distribution was assessed using a cell cycle assay kit (KeyGen BioTECH, Jiangsu, China). First, PC cells were incubated with 0.2 μM nocodazole for 24 hours to synchronize PC cells in the G2/M phase. Thereafter, the culture medium was removed and synchronized cells were treated with different concentrations (0, 20, 40, and 80 μM) of ST for 48 hours. Further, PC cells were collected, immobilized in 70% ethanol overnight at -20℃, washed with PBS two times, stained with propidium iodide reagent, and detected using a DeFLEX flow cytometer (Beckman, USA). The results were analyzed using Flwo JO (version 7.6.1).

### Western blotting

Total protein in PC cells was extracted using radioimmunoassay precipitation lysis buffer (Beyotime Biotechnology, Suzhou, China) containing phenylmethylsulfonyl fluoride (Servicebio, Wuhan, China). Protein concentrations in the samples were determined using the bicinchoninic acid method (Servicebio, Wuhan, China). Proteins were separated using 10% sodium dodecyl sulfate polyacrylamide gels (Meilune, Dalian, China)and thereafter, transferred to polyvinylidene fluoride membranes (Thermo Scientific, USA). The membranes were blocked with skim milk powder (Beyotime Biotechnology, Suzhou, China) and incubated with primary antibodies (GADD45A [1:500], Cat No. A1797, Abconal, China; CDK1 [1:500], Cat No. 19532-1-AP, Proteintech, China; CCNB1 [1:500], Cat No. 28603-1-AP, Proteintech, China; N-cadherin [1:500], Cat No. 22018-1-AP, Proteintech, China; E-cadherin [1:500], Cat No. 20874-1-AP, Proteintech, China; and β-actin [ACTB], 1:500; Cat No. 20536-1-AP, Proteintech) for 16 hours at 4℃. After washing twice with Tris-buffered saline containing 0.1% Tween‐20, the membranes were incubated with secondary antibody and visualized using an enhanced chemiluminescence reagent. ACTB was used as the loading control to calculate the relative protein expression.

### Wound healing assay

PC cells (6 × 10^5^) were cultured in the six-well plates. The cell monolayer was wounded using a 200-μL pipette tip. The cells were washed twice with PBS, floating cells were removed, and fresh medium with ST (0, 20, 40, and 80 μM) was added. The wound healing conditions were recorded from 0-24 hours using an optical microscope (magnification, 40×).

### Transwell assay

PC cells (2 × 10^4^ PC cells/well) were resuspended in 200 μL FBS-free DMEM and added to the upper transwell chamber (ThermoFisher Scientific, USA), which was pre-coated with Matrigel (ThermoFisher Scientific, USA) and 700 μL DMEM medium containing 10% FBS was added to the lower transwell chamber. Thereafter, PC cells were treated with different concentrations (0, 20, 40, and 80 μM) of ST. After 24 hours, the invaded cells were immobilized and stained with 0.5% crystal violet. Finally, an inverted microscope was used to photograph the invaded cells in each chamber. The number of cells in five random fields of each chamber was counted.

### Immunofluorescence analysis

PC cells were seeded into confocal dishes and treated with DMSO and ST. Thereafter, the cells were washed with PBS, immobilized with 4% formaldehyde, and permeabilized with 0.2% Triton X (Beyotime Biotechnology, Suzhou, China). The cells were blocked with 5% BSA and stained with anti-E-cadherin (1:200; Cat No. 20874-1-AP, Proteintech) antibody overnight at 4℃. Thereafter, cells were washed with PBS and incubated with CY3 label-secondary antibody. Cell nuclei were stained with 4,6-diamino-2-phenylindole. Finally, cells were visualized using a Ti2-U fluorescence microscope (Nikon, Japan) at 562 nm.

### RNA sequencing and bioinformatics analysis

Total RNA was extracted from PC cells treated with DMSO and ST for 24 hours using TRIzol Reagent (Thermo Fisher Scientific, USA) according to the manufacturer's instructions. RNA library construction and sample sequencing were performed at the Beijing Genomics Institute (BGI, Shenzhen, China). RNA quality was analyzed using a Bioanalyzer 2100 (Agilent). The BGISEQ-500 platform was used to sample the sequences. Original reads with >10% unknown beads and low-quality reads were removed. The count number was used as a unit to quantify the expression level of genes. Differentially expressed genes were analyzed using the EdgeR package (cut-off: LogFC > 1 and adjusted *P* < 0.05). The pathways of differentially expressed genes were analyzed using DAVID (https://david.ncifcrf.gov/home.jsp). Pathways with a P value of <0.05 were considered significant enrichment pathways. The protein-protein interaction (PPI) network of the proteins coded by differentially expressed genes was constructed using STRING (https://string-db.org/), in which A line indicates the connection between proteins and proteins with more lines indicate hubs.

### Reverse transcriptase quantitative PCR

Total RNA from PC cells treated with DMSO and those with ST was isolated using TRIzol reagent; thereafter, the complementary DNA (cDNA) of each sample was synthesized with 2-μg total RNA using PrimeScript 1st Strand cDNA Synthesis Kit (Takara, Japan). Reverse transcriptase quantitative PCR (RT-qPCR) was performed to detect gene expression with HieffTM qPCR SYBR Green Master Mix (Yeasen, Shanghai, China) using a CFX96 Touch fluorescence ration PCR instrument (Bio-Rad, USA). β-actin was used as the reference gene. The 2^-ΔΔ^Ct method was used to measure the relative fold change in the mRNA expression of a gene. The following primers were used for RT-qPCR: GADD45A Forward: 5'-GAGAGCAGAAGACCGAAAGGA-3',Reverse: 5'-CACAACACCACGTTATCGGG-3'; β‐actin Forward: 5'-TCAGAAGGATTCCTATGTGGGCGA-3', Reverse: 5'-TTTCTCCATGTCGTCCCAGTTGGT-3'.

### Immunohistochemical staining

The tumor tissues were immobilized, dehydrated, and embedded in paraffin; thereafter, the tumor tissue samples were cut into 2-μm sections and stored. Briefly, the IHC staining was performed as follows: sections were deparaffinized using xylene, rehydrated using alcohol, treated with the sodium citrate reagent for antigen restoration, blocked with H_2_O_2_ and BSA (Thermo Scientific, USA), and incubated with the primary antibodies (anti‐GADD45A antibody [1:100], Cat no. A1797; Abconal, China and anti‐KI67 antibody [1:100]; Cat No. 27309-1-AP, Proteintech, China) for 12 hours at 4°C. The next day, the sections were stained with horse radish peroxidase-conjugated goat anti-mouse and anti-rabbit secondary antibodies (Beyotime Biotechnology, Suzhou, China) for 2 hours. After incubation with diaminobenzidine substrate (Beyotime Biotechnology, Suzhou, China) and hematoxylin, immune signals were detected using an orthophoto microscope. Finally, the protein expression levels of the target genes were evaluated based on the sum of the intensity score (0: no staining; 1: weakly positive; 2: moderately positive; and 3: strongly positive) and the score for the proportion of positive cells (0: <1%; 1: 1%-33%; 2: 34%-66%; and 3: 67%-100%) using the Image-Pro Plus software (version 6.0; Media Cybernetics, Inc.).

### *In vivo* assay

Female BALB/c nude mice (age: 4 weeks) were purchased from the Animal Center of Guizhou Medical University. PANC-1 cells (2 × 10^6^ cells in 200 µL PBS) were subcutaneously injected into the right flank of each mouse. On day 9 after injection, mice with 40-60 mm^3^ sized tumors were randomly divided into the DMSO and ST treatment groups (5 mice per group). The ST group was intraperitoneally injected with 40 mg/kg ST every 3 days, whereas the DMSO group was intraperitoneally injected with 40 mg/kg DMSO (control) every 3 days. Tumor volume was measured every 3 days. After 18 days, all mice were euthanized and tumor tissues were extracted for further analysis. The animal experiment was approved by the Animal Ethics Committee of Guizhou Medical University.

### Statistical analysis

All statistical analyses were performed using SPSS software (version 19.0). Student's t-test and one-way analysis of variance with least significant difference t-test were used to analyze differences between the two groups and multiple groups, respectively. A *P* value of <0.5 was considered statistically significant.

## Results

### ST suppressed PC cell proliferation and induced PC cell G2/M phase arrest *in vitro*

**Figure 1:**
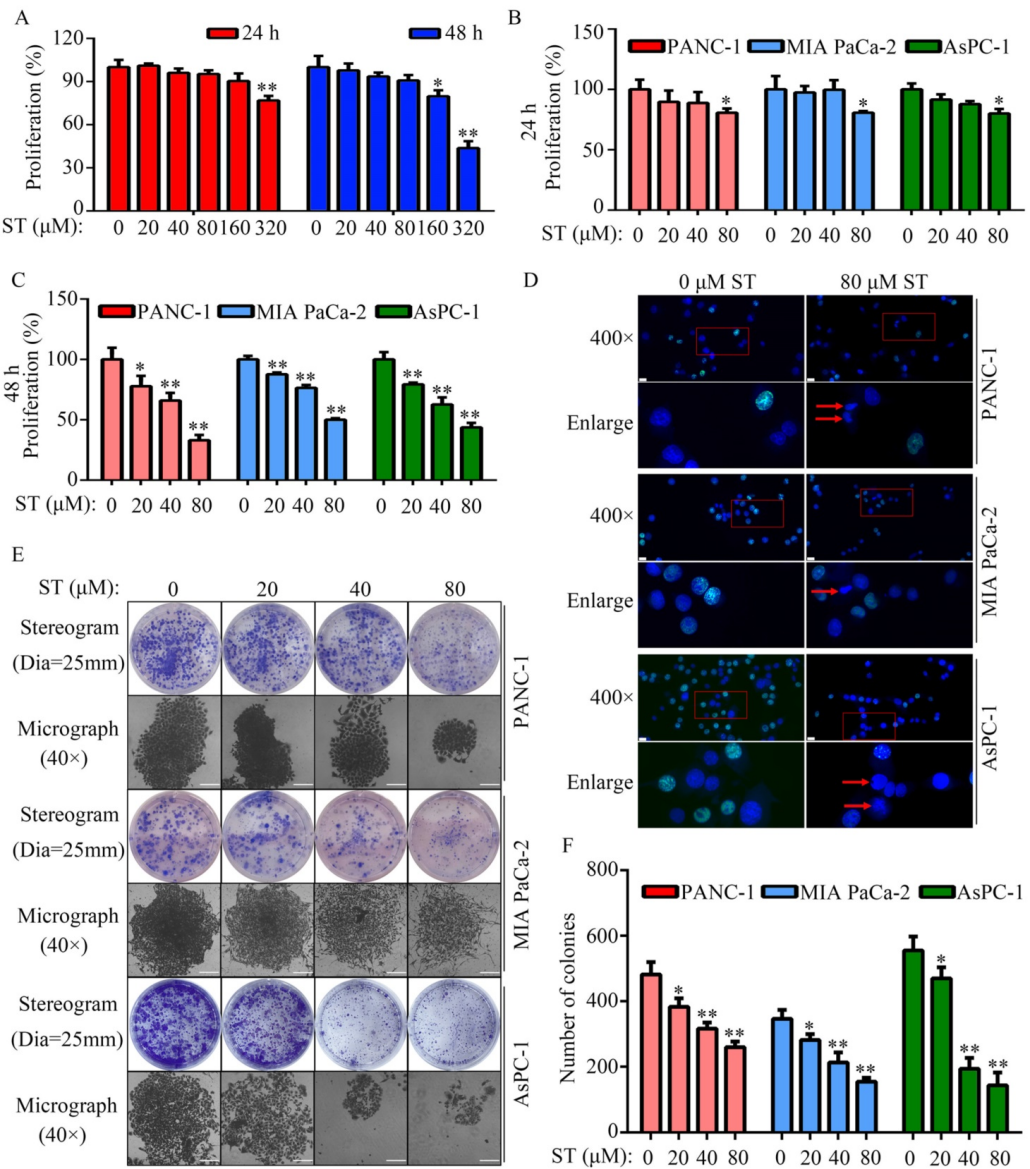
** ST inhibited PC cell proliferation *in vitro*. A.** Cell proliferation rate of normal human pancreatic epithelial cells treated with different concentrations (0, 20, 40, 80, 160 and 320 μM) of ST at 24 and 48 hours, as determined by the CCK-8 assay.** B-C.** PANC-1, MIA PaCa-2, and AsPC-1 cells were treated with different concentrations (0, 20, 40, and 80 μM) of ST. CCK-8 assay was performed to assess cell proliferation in each group at 24 and 48 hours. **D.** EdU assay was performed to detect the EdU-positive PC cells after treatment with DMSO and ST. White lines indicated 20 μm. Arrow indicates cell with nuclear atypia. **E-F.** Colony formation assay was performed to analyze the colony formation of PC cells treated with different concentrations (0, 20, 40, and 80 μM) of ST. Stereograms and representative micrographs were both exhibited, and white lines on micrographs indicate 100 μm.*, *P* < 0.05; **, *P* < 0.01.

**Figure 2:**
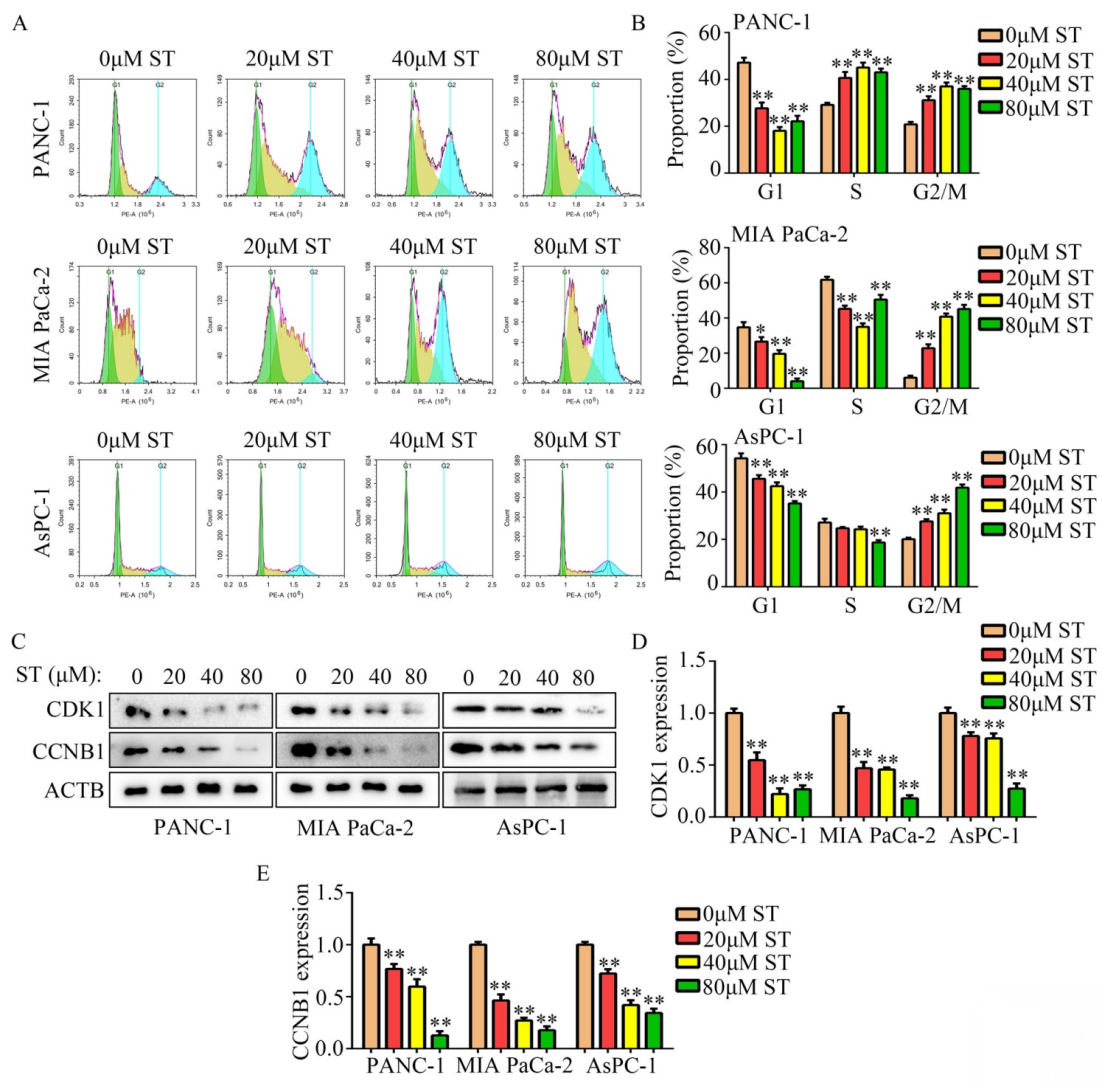
** ST induced PC cell arrest in the G2/M phase *in vitro*. A-B.** PANC-1, MIA PaCa-2, and AsPC-1 cells were treated with different concentrations (0, 20, 40, and 80 μM) of ST. Flow cytometry analysis was performed to determine the cell distribution in each group. **C-E.** Western blotting was performed to analyze the expression levels of CCNB1 and CDK1 in PC cells treated with different concentrations (0, 20, 40, and 80 μM) of ST. *, *P* < 0.05; **, *P* < 0.01.

Treatment of normal human pancreatic epithelial cells with different concentrations (0, 20, 40, 80, 160, and 320 μM) of ST revealed that 0, 20, 40, and 80 μM ST was not cytotoxic to normal human pancreatic epithelial cells at both 24 and 48 hours (*P* < 0.05, Figure 1A). To rule out nonspecific cytotoxicity, follow-up experiments were performed using 0, 20, 40, and 80 μM ST. PC cell lines (PANC-1, MIA PaCa-2, and AsPC-1) were treated with different concentrations (0, 20, 40, and 80 μM) of ST. CCK-8 assay revealed that ST markedly reduced the proliferation rate of PANC-1, MIA PaCa-2, and AsPC-1 cells at 24 and 48 hours. In addition, compared with other digestive system tumor cells, PC cells were more sensitive to ST (*P* < 0.05, Figure 1B-C and Table S1). The results of EdU assay revealed that ST treatment reduced the number of EdU-positive PANC-1 and MIA PaCa-2 cells. Analysis of the nuclear morphology revealed that ST significantly increased nuclear abnormalities in PC cells (*P* < 0.05, Figure 1D). Moreover, colony formation assays revealed that the colonies were lesser, smaller, and incompact in the ST treatment group compared with the DMSO treatment group (*P* < 0.05, Figure 1E-F). Furthermore, flow cytometry analysis revealed that the percentage of PANC-1 cells in the G2/M phase and S phase significantly increased, whereas that in G1 phase significantly decreased after ST treatment. Moreover, the percentage of MIA PaCa-2 and AsPC-1 cells in G2/M phase increased and those in G1 and S phase decreased after ST treatment (*P* < 0.01, Figure 2A-B). Western blot analysis revealed that ST treatment significantly decreased the expression of CDK1 and CCNB1, two biomarkers involved in the G2/M phase, in PANC-1, MIA PaCa-2, and AsPC-1 cells (*P* < 0.01, Figure 2C-E). These results indicate that ST significantly suppressed the proliferation of PC cells and induced G2/M phase arrest *in vitro*.

### ST reduced the motility and EMT of PC cells *in vitro*

**Figure 3:**
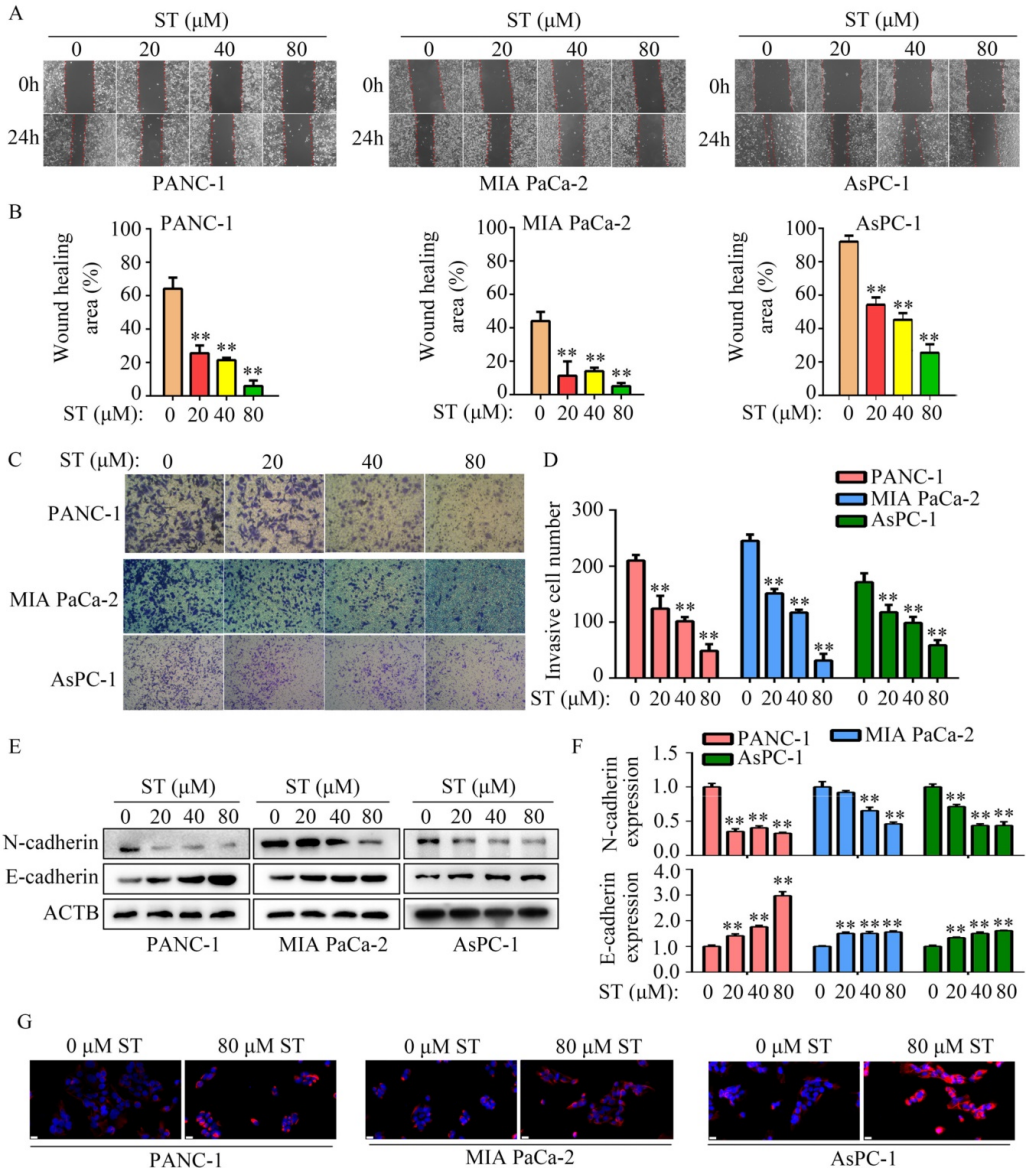
** ST suppressed the motility of PC cells *in vitro*. A-B.** Wound healing assay was performed to analyze the migration rate of PC cells treated with different concentrations (0, 20, 40, and 80 μM) of ST. **C-D.** Transwell assay was performed to analyze the invasion rate of PC cells treated with different concentrations (0, 20, 40, and 80 μM) of ST. **E**-**F.** Western blotting was performed to analyze the expression levels of E- and N-cadherins in PC cells treated with different concentrations (0, 20, 40, and 80 μM) of ST. **G.** Immunofluorescence was used to analyze the expression of E-cadherin in PC cells treated with DMSO and ST. White lines indicate 20 μm. **, *P* < 0.01.

Wound healing assays revealed that the migration capability of PANC-1, MIA PaCa-2, and AsPC-1 cells was reduced after ST treatment (*P* < 0.01, Figure 3A-B). Similarly, ST treatment markedly reduced the invasion ability of PANC-1 and MIA PaCa-2 cells (*P* < 0.01, Figure 3C-D). Previous studies have revealed that EMT is a key process during metastasis in several cancers such as PC 14,15. Hence, we investigated the expression of N- and E-cadherins in PC cells treated with ST. Western blotting analysis revealed that ST treatment prominently reduced the expression of N-cadherin (biomarker for mesenchymal phenotype) and upregulated the expression of E-cadherin (biomarker for epithelium phenotype; *P* < 0.01, Figure 3E-F). Similarly, immunofluorescence results revealed that E-cadherin expression was markedly elevated in the ST treatment group than in the DMSO treatment group (Figure 3G). Overall, these results indicate that ST reduced the motility and EMT in PC cells *in vitro*.

### GADD45A is a key target gene of ST

**Figure 4:**
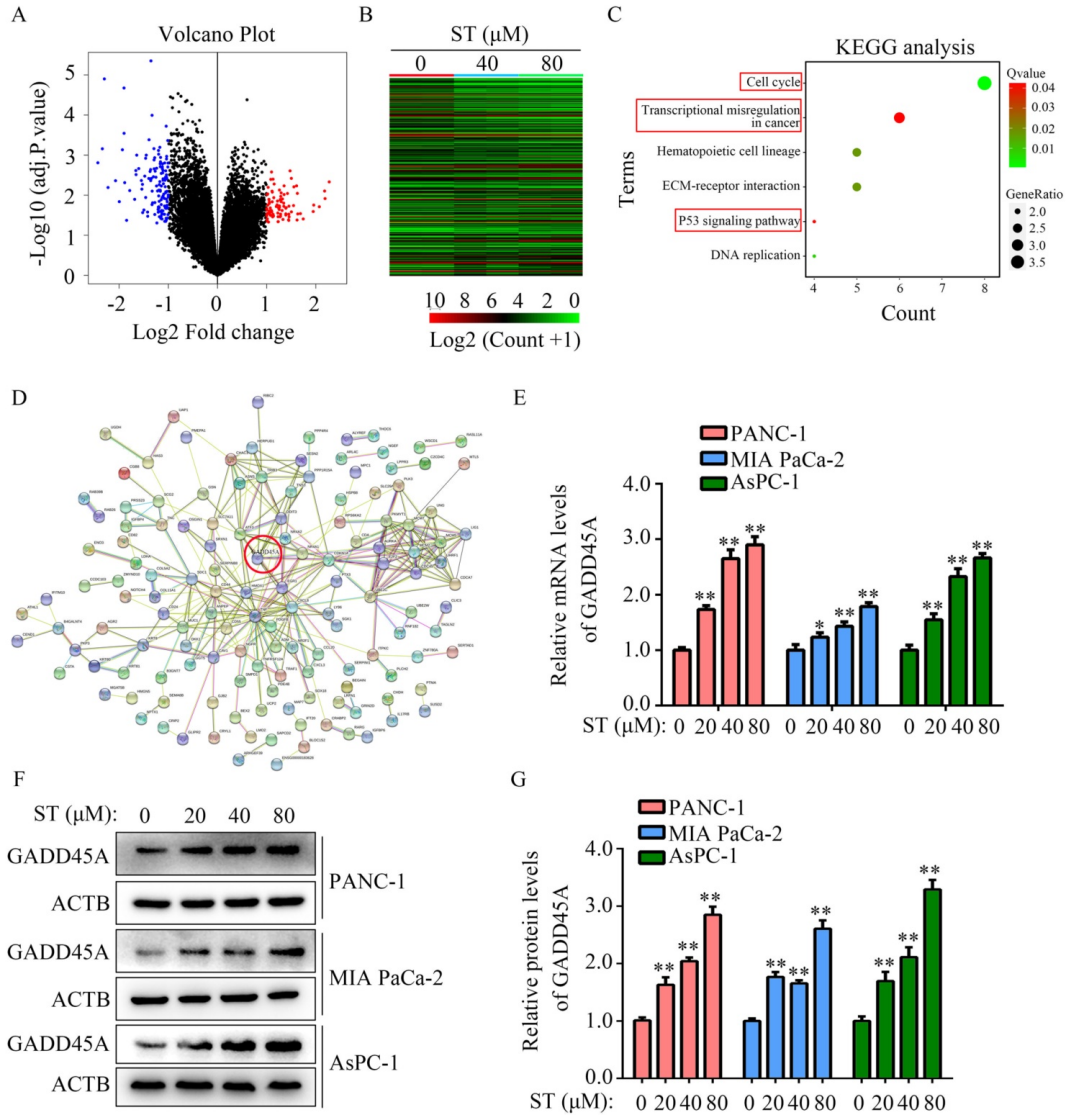
** GADD45A is the key target of ST. A-B.** Differentially expressed genes in PC cells treated with DMSO and ST were identified. **C.** KEGG analysis was performed to determine the pathways in which differentially expressed genes enriched. **D.** Protein-protein interaction network analysis was performed for differentially expressed genes. GADD45A had strong relationship with other proteins encoded by differentially expressed genes. **E.** RT-qPCR was performed to analyze the mRNA levels of GADD45A in PC cells treated with different concentrations (0, 20, 40, and 80 μM) of ST. **F-G.** Western blotting was performed to analyze the protein levels of GADD45A in PC cells treated with different concentrations (0, 20, 40, and 80 μM) of ST. *, *P* < 0.05; **, *P* < 0.01.

To uncover the molecular mechanism of ST in PC cells, high-throughput sequencing was performed. We found 136 genes were downregulated and 89 genes were upregulated in PANC-1 cells treated with ST (Figure 4A-B; Table S2). Kyoto Encyclopedia of Genes and Genomes (KEGG) analysis revealed that 225 differentially expressed genes were significantly enriched in transcriptional dysregulation in cancer, hematopoietic cell lineage, ECM receptor interaction, P53 signaling pathway, and cell cycle pathway (Figure 4C). PPI network analysis revealed that GADD45A had a strong relationship with proteins encoded by other differentially expressed genes (Figure 4D). Interestingly, we found that GADD45A was enriched in transcriptional dysregulation in cancer, P53 signaling pathway, and cell cycle pathway. Therefore, we considered that GADD45A may be an ST-regulated hub gene. RT-qPCR and Western blotting revealed that both the GADD45A mRNA and protein levels were elevated in PC cells treated with ST (*P* < 0.05, Figure 4E-G). Overall, these results suggest that GADD45A is the key target of ST.

### Silencing of GADD45A alleviated the inhibitory effects of ST on the proliferation and migration potential of PC cells

**Figure 5:**
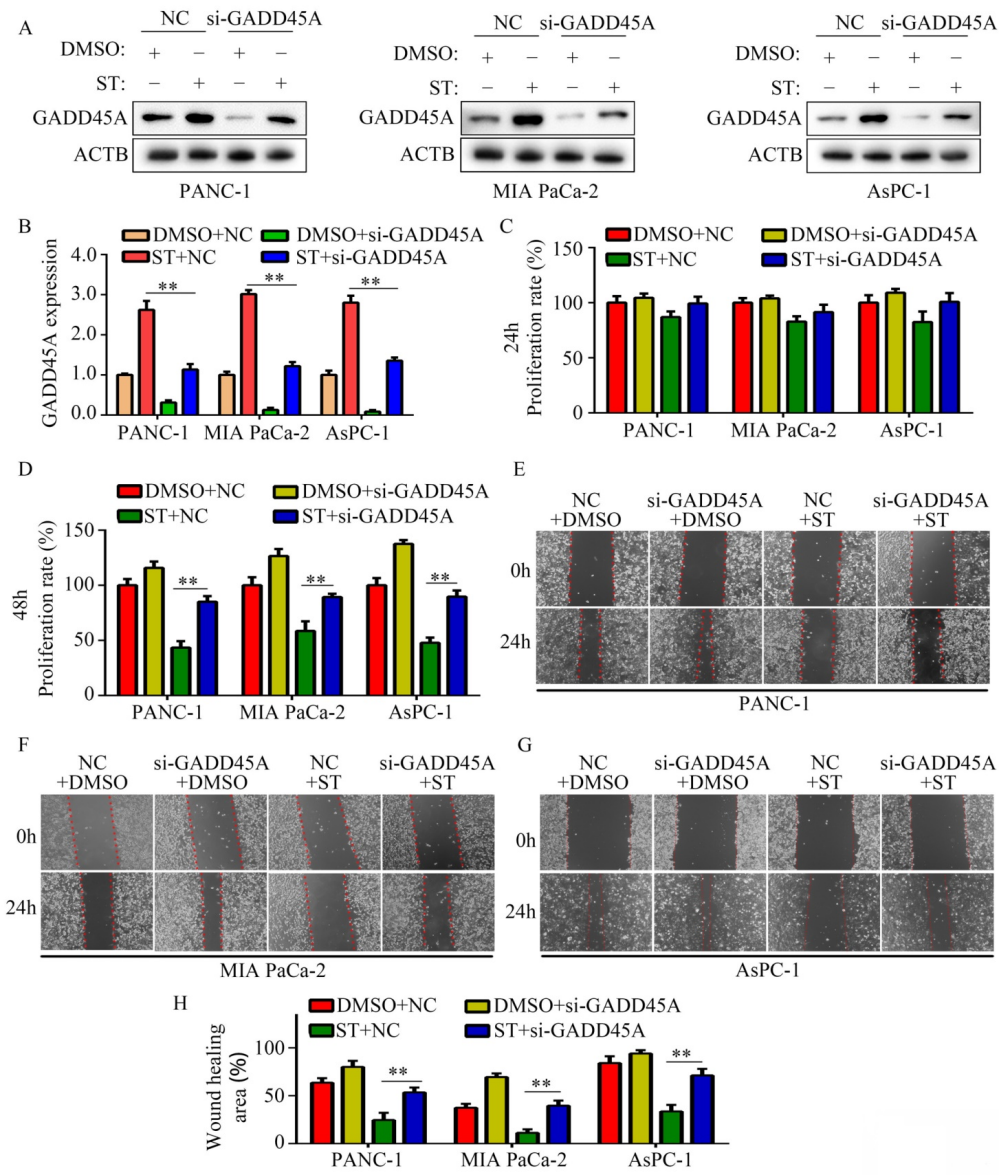
** Silencing of GADD45A reversed the inhibitory effects of ST on PC cells.** PC cells were treated with DMSO + NC siRNA, ST + NC siRNA, DMSO + GADD45A siRNA and ST + GADD45A siRNA. **A-B.** Western blotting was performed to analyze the expression levels of GADD45A in each group. **C-D.** CCK-8 assay was performed to analyze the proliferation rate of PC cells in each group at 24 and 48 hours. **E-H.** Wound healing assay was performed to analyze the migration rate of PC cells in each group. **, *P* < 0.05.

GADD45A has been recognized as a suppressor in several cancers. Hence, we hypothesized that GADD45A is involved in the biological functions induced by ST. Therefore, we transfected PC cells with GADD45A siRNAs prior to ST treatment (Figure 5A-B). CCK-8 assay revealed that GADD45A silencing alleviated the inhibitory effects of ST on PC cell proliferation at 48 hours, but not at 24 hours (Figure 5C-D). Wound healing assays revealed that GADD45A silencing significantly reversed the inhibitory effects of ST on the migration potential of PC cells (Figure 5E-H). These results indicate that GADD45A upregulation is involved in the ST-induced effects in PC.

### ST inhibited the proliferation and GADD45A expression in PC cells *in vivo*

**Figure 6:**
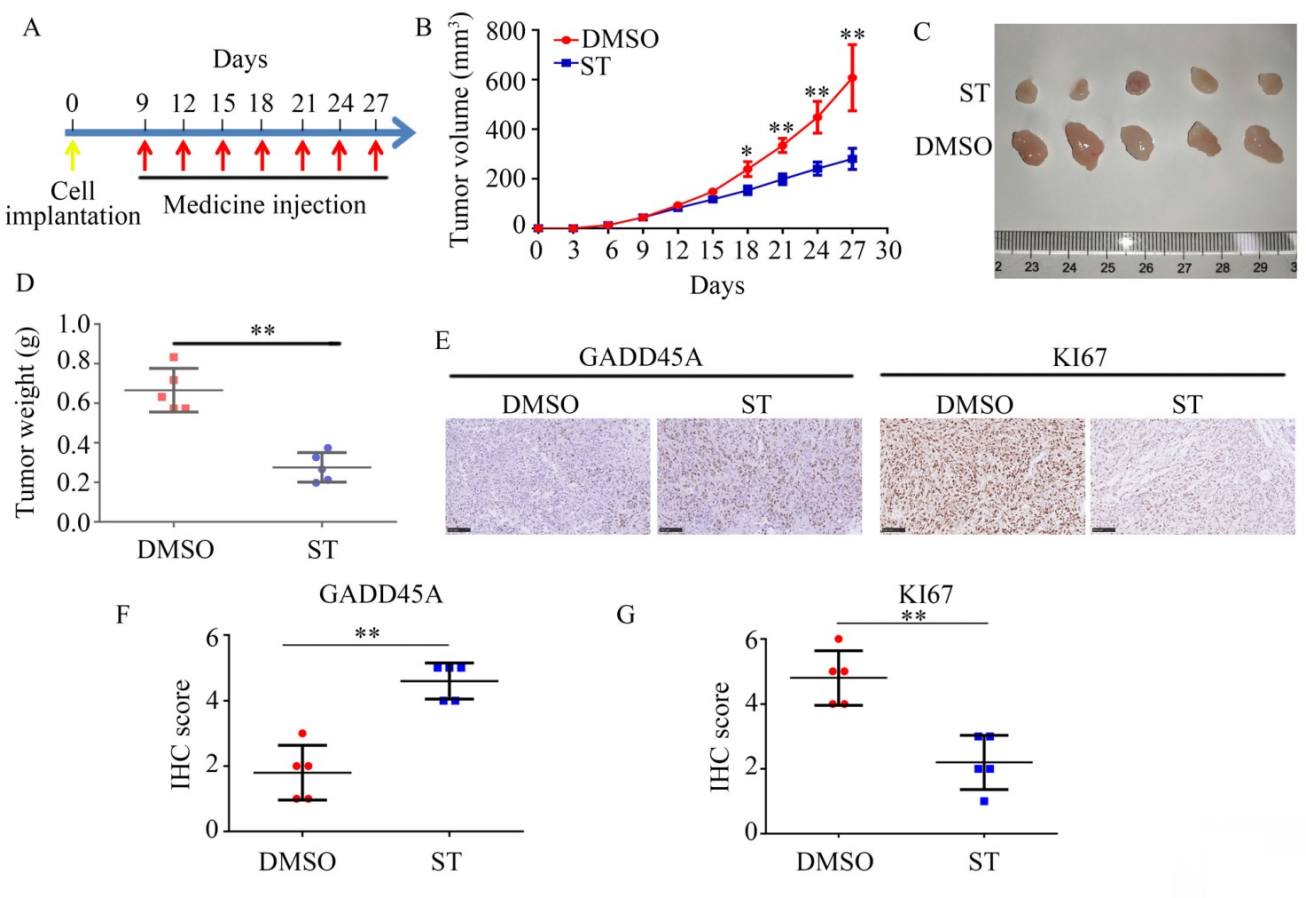
** ST repressed the proliferation rate of PANC-1 cells *in vivo* and increased the expression of GADD45A. A.** Schematic representation of animal experiments. **B-C.** Proliferation of tumor tissues in the DMSO and ST treatment groups. **D.** Tumor weight in the DMSO and ST treatment groups. **E-G.** Expression of GADD45A and KI67 in tumor tissues in the DMSO and ST treatment groups. Black lines in the left bottom indicate 100 μm. *, *P* < 0.05; **, *P* < 0.01.

To determine the effects of ST on PC cell proliferation *in vivo*, we used a xenograft tumor model. Compared with DMSO treatment, ST treatment decreased the proliferation rate of PC cells (*P* < 0.01, Figure 6A-C) and tumor weight (*P* < 0.01, Figure 6D) *in vivo*. Moreover, compared with DMSO treatment, ST treatment significantly decreased the expression of KI67 and increased the expression of GADD45A in tumor tissues (Figure 6E-G).

## Discussion

PC is a dreadful malignancy, considering the poor survival rate, decreased quality of life, and diagnostic and treatment difficulties 16 and distant metastasis at early stage 17. Excision and chemotherapy are the main therapeutic strategies for the early treatment of PC; however, few patients with PC benefit from these therapies because of severe side effects and unstoppable recurrence 18. Hence, uncovering the mechanisms underlying PC progression and development of novel therapeutic methods for the treatment of PC are essential.

An increasing body of evidence indicates that some natural products may have chemopreventive potential with low toxicity; moreover, there is a growing interest in the use of natural products as chemopreventive agents 19. To date, an increasing number of bioactive molecules derived from natural medicinal plants have been approved by the Food and Drug Administration as anticancer drugs 20. Fisetin, a natural flavonoid, has exhibited antitumor effects in various cancer models, including PC, by altering different signaling pathways 21. ST, a quaternary amine alkaloid in cruciferous plants, has been widely studied because of its various pharmacological effects. Previous studies have indicated that ST alleviates vascular endothelial dysfunction in hypertension by repressing the activation of the NLRP3 inflammasome 13 and protects against a prothrombotic state leading to inflammatory damage 22. Some studies have suggested that ST can be used as an effective natural compound for inhibiting the proliferation of Caco-2 cells through downregulation of P-glycoprotein 23. In the present study, we found that ST significantly inhibits PC cell proliferation, colony formation, and mobility and induces G2/M phase arrest at pharmacological doses. Interestingly, ST treatment arrested PANC-1, MIA PaCa-2, and AsPC-1 cells in the G2/M phase and increased the number of PANC-1 cells in the S phase and decreased the number of MIA PaCa-2 and AsPC-1 cells in the S phase. We speculate that this may be because of the different doubling times. Furthermore, the results of our study demonstrate that ST may be a novel and potential drug for PC treatment.

Next-generation sequencing (NGS), a massively parallel sequencing technology that allows the sequencing of targeted causal and candidate genes of various diseases, is used to analyze the order of nucleotides in entire genomes or targeted regions of DNA or RNA 24. In the current study, NGS revealed that the expression of 225 genes was significantly altered after ST treatment. A series of bioinformatics analyses revealed that GADD45Awas enriched in transcriptional dysregulation in cancer, P53 signaling pathway, and cell cycle pathway and had a strong relationship with other proteins coded by differentially expressed genes.

GADD45 family members (GADD45a, GADD45b, and GADD45g) act as stress sensors under physiological, environmental, and oncogenic stress 25. GADD45A proteins serve as tumor suppressors in various cancers and are connected to multiple cell signaling molecules; defects in the GADD45A protein are closely associated with the pathogenesis of malignancy 26,27. GADD45A expression is elevated in several cancer cells after treatment with anti-tumor drugs and is positively associated with drug efficiency. For example, previous studies have shown that ionizing radiation treatment induces GADD45A expression in cervical cancer and sensitizes cancer cells to radiotherapy 28. Mo *et al*. 29 revealed that the activation of the P53/GADD45A pathway reduces the proliferation and metastasis of breast cancer cells. These studies highlight the induction of GADD45A expression as a cancer therapeutic strategy. In the present study, we found that GADD45A expression was increased in PC cells after ST treatment *in vitro* and* in vivo*. The inhibition of GADD45A expression markedly suppressed the effects of ST on PC cell proliferation and mobility. These results indicate that GADD45A is involved in the biological process induced by ST. However, the specific anti-PC mechanism of ST is complicated. Our study only considered GADD45A as a pointcut to study.

In conclusion, ST significantly affected PC cell proliferation and mobility by upregulating GADD45A. ST may be a novel and effective drug for PC treatment.

## Supplementary Material

Supplementary tables.

## Abbreviations

CCK-8: cell count kit-8
EdU: 5-Ethynyl-2'-deoxyuridine
FBS: fetal bovine serum
GADD45A: growth arrest and DNA damage inducible alpha
PBS: phosphate buffer saline
PC: Pancreatic cancer
RT-qPCR: real-time quantitative polymerase chain reaction
ST: Sinapine thiocyanate
