# Supplementary material for: Sinapine Thiocyanate Inhibits the Proliferation and Mobility of Pancreatic Cancer Cells by Up-Regulating GADD45A
Source: J Cancer. 2022 Jan 24;13(4):1229–40. doi: 10.7150/jca.65212 (PMC8899366; doi:10.7150/jca.65212)
Supplement: Supplementary file 1 — Supplementary tables. [file jcav13p1229s1.pdf]

Table S1 Proliferation rate (mean ± standard deviation) of other cancer cells (HepG2, HuH7, Hep3B, TFK-1, and RBE) after ST treatment

Cell lines	24 h proliferation rate（%）				48 h proliferation rate（%）			
	0 μM	20 μM	40 μM	80 μM	0 μM	20 μM	40 μM	80 μM
Huh7	100.0 ± 6.2	96.4 ± 4.2	93.5 ± 6.8	85.2 ± 6.8	100.0 ± 8.3	92.2 ± 4.6	84.5 ± 6.2	75.4 ± 3.1
Hep3B	100.0 ± 9.3	99.4 ± 6.4	95.9 ± 4.8	90.1 ± 3.4	100.0 ± 5.2	94.4 ± 2.4	84.1 ± 7.1	71.9 ± 4.2
HepG2	100.0 ± 7.4	103.4 ± 6.5	97.4 ± 4.1	93.2 ± 3.9	100.0 ± 6.7	91.3 ± 3.9	83.4 ± 6.2	72.3 ± 4.7
TFK-1	100.0 ± 4.3	99.1 ± 3.8	99.4 ± 5.5	92.1 ± 3.1	100.0 ± 5.8	96.5 ± 4.3	90.6 ± 5.5	81.2 ± 4.8
RBE	100.0 ± 3.1	98.2 ± 2.9	96.5 ± 3.7	90.2 ± 6.1	100.0 ± 4.8	95.1 ± 2.2	91.1 ±4.3	82.9 ± 6.2

Table S2 Differentially expressed genes between the DMSO and ST treatment groups

ID	logFC	AveExpr	t	P.Value	B
CCL20	2.27204	4.311156	4.731326	0.004674	-1.54879
DDIT3	2.187731	6.453646	3.765376	0.012142	-2.51077
SESN2	2.178722	5.615307	4.186065	0.00788	-2.07191
BEX2	2.070522	2.69016	3.335069	0.019437	-2.9918
CSTA	1.956998	1.998668	3.420244	0.017668	-2.89405
VSTM1	1.947008	2.541876	3.095917	0.025567	-3.27266
CXCL8	1.74381	4.30929	2.737948	0.03918	-3.70917
ANKRD1	1.686594	1.697251	2.993806	0.02882	-3.39532
H2AC8	1.632253	3.318004	2.979161	0.029323	-3.41304
SMPD1	1.624774	5.576406	4.051686	0.009021	-2.2087
H4C8	1.618538	2.351247	2.687461	0.041675	-3.77209
H2BC4	1.608081	4.496623	4.492228	0.005847	-1.77195
LMO2	1.553448	2.390835	4.430256	0.006204	-1.83137
OSGIN1	1.545793	4.451013	3.395771	0.018157	-2.92201
SERPINB8	1.502846	2.693821	3.078757	0.026084	-3.29317
CHAC1	1.502082	5.929347	4.958398	0.003805	-1.34556
GLIPR2	1.475819	4.18295	5.457937	0.002476	-0.92693
ETFRF1	1.456551	3.840797	2.836702	0.03476	-3.587
MXD4	1.452658	4.864521	3.36494	0.018795	-2.95738
HMOX1	1.450363	6.716286	2.966155	0.029779	-3.42881
EGR1	1.448895	2.616453	2.668849	0.042638	-3.79536
ABTB1	1.444821	3.130521	2.695912	0.041246	-3.76154
TRIB3	1.422936	6.821997	4.04716	0.009062	-2.21336
WSCD1	1.396538	1.598425	3.289097	0.020475	-3.04507
SERPINI1	1.389437	2.813088	2.923148	0.03134	-3.4811
NGFR	1.389194	1.988147	3.258997	0.021187	-3.08013
SERTAD1	1.389142	5.284962	2.984347	0.029144	-3.40677
CHMP4C	1.354242	2.86117	4.491951	0.005849	-1.77222
EVI5L	1.354228	3.745616	3.984707	0.009659	-2.27807
HMGN5	1.347937	2.460601	3.359383	0.018913	-2.96377
METTL7B	1.34612	2.954947	3.522399	0.015781	-2.77845
ZNF419	1.331389	3.686934	2.714416	0.040322	-3.73846

SRXN1	1.321169	6.307931	3.743079	0.012433	-2.5349
CGB8	1.314429	1.532193	3.14671	0.024103	-3.21224
AAMDC	1.295457	6.599841	3.595235	0.014574	-2.69712
CDRT4	1.294468	1.322854	4.079093	0.008773	-2.18054
CBWD5	1.287134	5.136673	3.884463	0.010714	-2.38337
FAM229B	1.282957	7.370685	2.968053	0.029712	-3.42651
LOC102724770	1.282257	5.652646	2.87515	0.033191	-3.53978
RAB39B	1.276772	2.293279	3.715205	0.012808	-2.56519
SLC7A11	1.275929	2.27447	3.690016	0.013157	-2.59268
PPP1R15A	1.27411	5.546317	2.842634	0.034513	-3.5797
HSPB8	1.261788	3.368188	3.307572	0.02005	-3.02362
LEPROT	1.260082	4.778096	3.375179	0.01858	-2.94562
HERPUD1	1.260035	4.924656	4.24318	0.007445	-2.01472
H2AC7	1.254873	4.058675	3.196224	0.022765	-3.15374
TM4SF19	1.243114	5.016856	3.270985	0.0209	-3.06615
LY96	1.235657	1.910506	2.613836	0.045631	-3.86436
ZNF780A	1.231768	3.856956	2.84349	0.034477	-3.57865
ZNF226	1.230814	4.08568	2.610555	0.045816	-3.86848
MORN2	1.226653	6.131108	3.021007	0.027911	-3.3625
TNF	1.219455	2.518105	3.630551	0.014027	-2.65802
CBWD6	1.212568	4.122939	3.432797	0.017423	-2.87975
CDKN1A	1.195811	7.495157	3.349066	0.019133	-2.97566
CRYL1	1.152383	5.386176	4.600939	0.005276	-1.6693
CXCL3	1.14499	2.608261	3.812165	0.011557	-2.46042
PLK3	1.13863	3.509639	2.879582	0.033015	-3.53435
SLC17A7	1.129402	1.90819	4.903689	0.003996	-1.39376
CLGN	1.128848	2.062467	2.959076	0.03003	-3.4374
ATF3	1.121194	4.511578	2.836129	0.034784	-3.58771
H2AC6	1.114329	5.655435	4.12065	0.008413	-2.1381
PPP4R4	1.112749	2.10968	3.590102	0.014656	-2.70283
TSPAN33	1.109406	3.701713	4.177789	0.007945	-2.08024
SEL1L3	1.1042	1.050471	3.076651	0.026148	-3.29569
ASNS	1.099268	7.209186	5.380923	0.00264	-0.98901
LMF1	1.098576	2.706633	3.140183	0.024286	-3.21999
TRAF1	1.083046	3.697289	4.890819	0.004043	-1.40517
IFITM10	1.082477	2.233134	3.084717	0.025903	-3.28604
GADD45A	1.077276	4.977931	2.911888	0.031764	-3.49484
MPC1	1.072754	4.626306	3.271682	0.020884	-3.06534
H2AC21	1.065947	0.710631	2.958728	0.030042	-3.43782
BLOC1S2	1.057003	3.321991	3.123823	0.024751	-3.23942
PTX3	1.054477	2.413914	3.506123	0.016065	-2.79675
H2BC5	1.053763	5.539526	3.572553	0.014938	-2.72235
CALHM6	1.052959	1.207863	3.090924	0.025716	-3.27862
UAP1	1.048281	4.536363	3.698322	0.013041	-2.58361
TUBE1	1.047423	4.018869	3.72191	0.012716	-2.55789
IFT20	1.037429	4.717317	2.592899	0.046829	-3.8907
PLAAT3	1.030112	4.931274	4.40068	0.006384	-1.85995
TNFRSF12A	1.022102	9.685506	3.417195	0.017728	-2.89753
CD82	1.019314	3.992295	4.022609	0.009292	-2.23872
UBE2W	1.013196	2.139976	3.007201	0.028368	-3.37914

NDUFA4	1.012714	8.144502	2.741806	0.038996	-3.70438
ASDURF	1.011897	3.142381	2.638267	0.044274	-3.83368
SMIM29	1.01161	6.744834	3.982268	0.009683	-2.28061
ITPKC	1.006387	3.724902	2.965336	0.029807	-3.4298
ABHD16A	1.006095	5.209587	3.177992	0.023248	-3.17524
DHRS2	1.002312	2.128792	3.242408	0.021592	-3.09952
RPS6KA2	1.001818	1.342085	2.8879	0.032688	-3.52416
COL11A1	-1.0017	1.445507	-3.96409	0.009866	-2.29959
PALM	-1.00277	3.562673	-3.85521	0.011046	-2.41444
RAP1GAP2	-1.00611	2.829393	-6.02056	0.001578	-0.49897
PSPC1	-1.00735	3.986919	-2.94182	0.030651	-3.45837
ADM	-1.01037	4.648584	-5.73132	0.001981	-0.71349
C19orf25	-1.01179	4.664937	-3.27454	0.020816	-3.06201
CD24	-1.01195	1.288419	-7.89605	0.000434	0.650687
MCM5	-1.01619	5.521586	-2.77768	0.037332	-3.65988
NRM	-1.02299	4.866351	-3.59685	0.014548	-2.69533
LRFN1	-1.02612	4.651136	-3.41016	0.017868	-2.90556
PGGHG	-1.02721	2.305485	-4.16629	0.008037	-2.09183
RARG	-1.02966	4.516652	-4.93763	0.003876	-1.36379
CHI3L2	-1.03405	1.502807	-6.53334	0.001076	-0.14532
ARL4C	-1.03583	3.551251	-5.02468	0.003588	-1.28779
HAS3	-1.03639	1.340574	-6.32855	0.00125	-0.28262
CCDC51	-1.03805	4.680543	-3.73216	0.012578	-2.54675
PMEPA1	-1.03861	3.508569	-5.18925	0.003108	-1.14735
MCM2	-1.04331	5.326142	-2.66507	0.042837	-3.80009
ADRA1B	-1.04356	4.918809	-9.3447	0.00019	1.313178
SH3BP1	-1.04374	2.270665	-3.37636	0.018556	-2.94427
GATD3A	-1.04409	1.932923	-2.77237	0.037573	-3.66646
ARHGEF16	-1.0459	3.263346	-3.25086	0.021385	-3.08964
THOC6	-1.05134	5.100222	-4.14137	0.00824	-2.11706
GSN	-1.05316	7.758712	-5.45866	0.002474	-0.92635
UNG	-1.05424	5.334742	-2.62474	0.04502	-3.85065
MTMR11	-1.05513	2.149536	-4.0877	0.008697	-2.17173
GGT5	-1.05913	1.166121	-5.02894	0.003574	-1.28411
GJB2	-1.05973	2.718326	-3.27037	0.020915	-3.06687
CCDC106	-1.06386	5.823817	-4.43845	0.006156	-1.82347
NOTCH4	-1.06702	1.059464	-4.8369	0.004244	-1.45326
PTMA	-1.07393	9.25978	-2.62811	0.044833	-3.84642
OOEP	-1.07404	2.506014	-4.86825	0.004126	-1.42524
IGFBP6	-1.07415	3.712377	-6.02735	0.00157	-0.49407
NGEF	-1.07735	3.90835	-4.8848	0.004064	-1.41051
LIG1	-1.07768	3.894903	-2.96192	0.029929	-3.43395
C2CD4C	-1.07782	1.239508	-3.77364	0.012036	-2.50184
NR4A2	-1.07871	1.428975	-3.37234	0.01864	-2.94888
SLC29A1	-1.08144	6.749359	-3.7724	0.012052	-2.50318
CLIC3	-1.08647	2.741221	-2.96489	0.029823	-3.43034
ALYREF	-1.09235	8.0976	-4.01241	0.009389	-2.24928
CHD5	-1.09962	2.944624	-4.61961	0.005185	-1.65187
CTXN1	-1.10239	5.687087	-4.87538	0.004099	-1.41889
ISYNA1	-1.10549	7.164466	-3.91543	0.010375	-2.35065

P2RX5	-1.10711	2.75017	-4.92688	0.003914	-1.37327
TAGLN2	-1.1081	9.627121	-4.83672	0.004245	-1.45342
TNFAIP8L1	-1.10855	2.464802	-5.48782	0.002415	-0.90308
COL5A2	-1.10921	3.991316	-3.12941	0.024591	-3.23278
CRIP2	-1.11444	8.004184	-5.32119	0.002777	-1.03776
CCDC103	-1.11701	1.244762	-5.96031	0.001653	-0.54273
MYEOV	-1.12153	6.298107	-5.60148	0.0022	-0.81353
ZMYND10	-1.12292	1.381709	-5.73944	0.001968	-0.70731
TM4SF18	-1.12616	3.227474	-3.82454	0.011407	-2.44716
UBE2C	-1.12904	7.956073	-2.73797	0.039179	-3.70915
CAV1	-1.13034	5.518068	-2.99163	0.028894	-3.39796
E2F2	-1.13382	2.135161	-2.9625	0.029908	-3.43324
ARHGEF39	-1.13459	4.404863	-3.14444	0.024166	-3.21494
MUC1	-1.14182	2.092224	-4.47959	0.005918	-1.78402
TESMIN	-1.14256	1.598101	-5.28656	0.002859	-1.06627
CITED2	-1.14367	5.337265	-3.44691	0.017152	-2.8637
TYMSOS	-1.14394	2.595943	-3.70303	0.012975	-2.57847
CDC45	-1.14601	4.031252	-2.86554	0.033576	-3.55157
PKMYT1	-1.15081	5.446651	-2.76225	0.038038	-3.679
HCFC1R1	-1.15765	6.523417	-6.45083	0.001143	-0.20003
PLEKHN1	-1.15939	1.7824	-6.11291	0.00147	-0.43282
EFEMP1	-1.15981	2.367872	-3.10387	0.025331	-3.26317
CD55	-1.16245	4.886129	-5.75206	0.001948	-0.69772
SAPCD2	-1.16313	3.460809	-4.17717	0.00795	-2.08086
MMACHC	-1.1643	2.781248	-4.23245	0.007525	-2.02542
PRSS23	-1.16592	6.321051	-5.96945	0.001642	-0.53606
FAM43A	-1.17277	3.966397	-7.28778	0.00064	0.319047
PDGFB	-1.17305	4.395861	-4.749	0.004599	-1.53267
MCM7	-1.18199	7.059317	-2.8429	0.034502	-3.57938
GRIN2D	-1.18482	1.773329	-4.56158	0.005475	-1.70624
DIPK1B	-1.18568	3.866274	-3.29986	0.020226	-3.03256
RIBC2	-1.18626	1.416754	-3.61684	0.014236	-2.67317
MGAT5B	-1.19154	3.655404	-4.23528	0.007504	-2.02259
CD44	-1.19282	7.973285	-3.2568	0.02124	-3.0827
CEND1	-1.19382	4.227492	-3.33378	0.019466	-2.9933
RAB26	-1.19535	4.464609	-3.91126	0.01042	-2.35505
IRX3	-1.19673	3.052498	-6.70786	0.00095	-0.03225
NPTX1	-1.19934	2.097183	-7.01817	0.000766	0.160249
CTRL	-1.2025	2.087279	-3.31793	0.019817	-3.01162
PDE4B	-1.2046	1.853594	-3.06388	0.026541	-3.31097
NR2F1	-1.20822	2.940958	-3.20089	0.022644	-3.14825
RNF182	-1.22033	3.164644	-4.18861	0.00786	-2.06934
AURKA	-1.23318	5.425851	-2.55066	0.049352	-3.94396
SOX18	-1.23828	1.802286	-5.64319	0.002126	-0.78113
SDC1	-1.24043	5.280592	-4.48388	0.005894	-1.77992
PKP3	-1.25095	4.925351	-5.02996	0.003571	-1.28323
UGDH	-1.26755	3.219181	-5.43684	0.002519	-0.94385
WDR4	-1.27418	4.204124	-3.55069	0.015299	-2.74675
SLC25A10	-1.2759	6.405418	-2.95143	0.030303	-3.44668
RNF208	-1.27759	2.697518	-6.42611	0.001164	-0.21657

CHDH	-1.28844	1.374753	-5.59942	0.002203	-0.81514
PLPPR3	-1.28998	3.126725	-6.25653	0.001319	-0.33214
BEGAIN	-1.29252	3.070641	-7.17026	0.000692	0.250764
ENO3	-1.30264	2.643952	-5.06171	0.003473	-1.25583
ANPEP	-1.32111	6.257453	-3.53769	0.015518	-2.7613
MAP7	-1.32909	2.575488	-4.18131	0.007917	-2.07669
TP53I11	-1.33136	3.902451	-10.592	0.000102	1.76805
CPNE7	-1.33179	2.860568	-3.44473	0.017193	-2.86618
SCARA3	-1.3452	2.718511	-4.9531	0.003823	-1.35021
SUSD2	-1.35325	2.624765	-19.5671	4.47E-06	3.403991
PPP1R14A	-1.35785	9.286301	-4.81526	0.004328	-1.47269
SYT12	-1.35939	2.914845	-5.54752	0.002299	-0.85581
LOC112694756	-1.37193	4.966042	-5.41473	0.002566	-0.96165
SCG2	-1.39256	0.936188	-7.97205	0.000414	0.689697
UCP2	-1.39676	4.60506	-4.9517	0.003828	-1.35143
UHRF1	-1.41948	3.486629	-2.86709	0.033513	-3.54966
SEMA6B	-1.42259	3.421784	-3.06841	0.026401	-3.30556
TNC	-1.4486	1.755119	-6.63311	0.001002	-0.08024
TNS3	-1.4604	3.172736	-3.82227	0.011434	-2.44959
KRT8	-1.46112	8.21251	-6.38232	0.001202	-0.24608
CDA	-1.47956	4.523268	-5.10268	0.00335	-1.22071
LDHA	-1.49667	9.511987	-3.17103	0.023435	-3.18346
DKK1	-1.52173	5.004414	-3.0402	0.027288	-3.3394
CDCA7	-1.52599	2.207915	-4.58035	0.005379	-1.68859
SGK1	-1.53856	4.194493	-4.29627	0.007066	-1.96207
YBX2	-1.54847	4.207082	-4.82069	0.004307	-1.46781
NR4A1	-1.60199	3.256992	-5.61727	0.002172	-0.80124
RASL11A	-1.64321	3.33426	-6.63274	0.001002	-0.08048
KRT81	-1.65647	1.238555	-5.20397	0.003069	-1.135
CCN5	-1.66042	2.776224	-5.48063	0.002429	-0.90881
MISP	-1.68522	3.815992	-3.72258	0.012707	-2.55716
SECTM1	-1.78504	4.706779	-4.21323	0.00767	-2.04464
AGR2	-1.84904	5.045295	-2.67296	0.042424	-3.79022
KRT80	-1.88776	5.289995	-4.68619	0.004873	-1.59019
KCNIP3	-1.90049	2.140015	-8.5975	0.000286	0.991937
B3GNT7	-1.90174	2.299632	-14.4499	2.12E-05	2.724278
STMN3	-1.93409	3.24631	-7.07804	0.000736	0.196175
B4GALNT4	-1.99716	2.881024	-3.59851	0.014522	-2.69348
IL17RB	-2.07372	1.673767	-4.8094	0.004352	-1.47797
PLCH2	-2.22619	3.308441	-4.39158	0.00644	-1.86878
PADI2	-2.30089	1.926317	-15.9815	1.26E-05	2.977732
CRABP2	-2.34175	5.535182	-7.15873	0.000697	0.243991
IGFBP4	-2.43333	5.940887	-6.04017	0.001554	-0.48483
